# Role of visceral fat on postoperative complications and relapse in patients with Crohn's disease after ileocecal resection: Is it overrated?

**DOI:** 10.1007/s00384-023-04586-4

**Published:** 2024-01-19

**Authors:** Christian Hans Wolfgang Schineis, Ioannis Pozios, Katharina Boubaris, Benjamin Weixler, Carsten Kamphues, Georgios Antonios Margonis, Martin Ernst Kreis, Rahel Maria Strobel, Katharina Beyer, Claudia Seifarth, Jan Luitjens, David Kaufmann, Johannes Christian Lauscher

**Affiliations:** 1 Department of General and Visceral Surgery, Charité-Universitätsmedizin Berlin, Corporate Member of Freie Universität Berlin and Humboldt-Universität zu Berlin, 12203 Berlin, Germany; 2 Department of General- and Visceral Surgery, Schloßparkklinik, Berlin, Germany; 3 Department of Surgery, Memorial Sloan Kettering Cancer Center, New York, NY USA; 4 Department of Diagnostic and Interventional Radiology and Neuroradiology, University Hospital Augsburg, Augsburg, Germany

**Keywords:** Visceral fat, Crohn’s disease, Ileocecal resection, Anastomotic leakage, Recurrence, Postoperative outcome, Complications, MRI

## Abstract

**Introduction:**

The role of visceral fat in disease development, particularly in Crohn´s disease (CD), is significant. However, its preoperative prognostic value for postoperative complications and CD relapse after ileocecal resection (ICR) remains unknown. This study aims to assess the predictive potential of preoperatively measured visceral and subcutaneous fat in postoperative complications and CD recurrence using magnetic resonance imaging (MRI). The primary endpoint was postoperative anastomotic leakage of the ileocolonic anastomosis, with secondary endpoints evaluating postoperative complications according to the Clavien Dindo classification and CD recurrence at the anastomosis.

**Methods:**

We conducted a retrospective analysis of 347 CD patients who underwent ICR at our tertiary referral center between 2010 and 2020. We included 223 patients with high-quality preoperative MRI scans, recording demographics, postoperative outcomes, and CD recurrence rates at the anastomosis. To assess adipose tissue distribution, we measured total fat area (TFA), visceral fat area (VFA), subcutaneous fat area (SFA), and abdominal circumference (AC) at the lumbar 3 (L3) level using MRI cross-sectional images. Ratios of these values were calculated.

**Results:**

None of the radiological variables showed an association with anastomotic leakage (TFA p = 0.932, VFA p = 0.982, SFA p = 0.951, SFA/TFA p = 0.422, VFA/TFA p = 0.422), postoperative complications, or CD recurrence (TFA p = 0.264, VFA p = 0.916, SFA p = 0.103, SFA/TFA p = 0.059, VFA/TFA p = 0.059).

**Conclusions:**

Radiological visceral obesity variables were associated with postoperative outcomes or clinical recurrence in CD patients undergoing ICR. Preoperative measurement of visceral fat measurement is not specific for predicting postoperative complications or CD relapse.

## Introduction

Crohn´s disease (CD) is a chronic inflammatory disorder affecting the gastrointestinal tract, with its complete pathophysiology still not fully understood. Emerging evidence suggests that visceral obesity plays a significant role in the pathogenesis of inflammatory bowel disease (IBD) [1, 2]. Visceral adipose tissue functions as a metabolically active organ, exhibiting hormonal and immune-related activities involved in lipid storage and immune response to gut microbiota. Excessive secretion of pro-inflammatory cytokines and adipokines by visceral fat may trigger acute intestinal inflammation, leading to chronic inflammation [1, 2]. Chronic inflammation, in turn, can increase visceral adipose tissue volume due to the inflammatory reaction. While medical treatment remains the primary approach for CD management, almost half of the patients require surgical intervention within 10 years of diagnosis and approximately 30% of them may need further surgical treatment [3]. Perioperative management of CD patients presents challenges due to the complex nature of CD surgery and its associated postoperative course. Various factors contribute to postoperative complications, including malnutrition, medical therapy, severity of CD, extend of bowel resection, anemia and others, leading to anastomotic leakage, postoperative bowel paralysis, surgical site infections, and more [4]. Hence, optimizing patient conditions and preoperative risk stratification are essential to minimize complications.

Body composition, often characterized solely by obesity based on a high body mass index (BMI), remains a contentious factor influencing surgical outcomes and complication rates in colorectal cancer surgery [5, 6]. Obesity, typically defined as a BMI ≥ 30 kg/m [2], is known to contribute to extended operating times and higher complication rates in colorectal surgery [6]. However, several studies have reported conflicting results, suggesting no significant impact of a high BMI on surgical outcomes [7, 8]. In the context of CD, obesity is not commonly considered a primary concern, as many patients experience weight loss due to intestinal inflammation and stenosis, leading to malnutrition and cachexia rather than excessive visceral obesity. While BMI serves as a readily accessible marker for assessing total adipose tissue accumulation, it fails to efficiently demonstrate fat distribution, particularly the ratio between visceral and subcutaneous fat [9]. Visceral obesity is recognized as an inflammatory state, with adipose tissue releasing cytokines and triggering immune reactions that perpetuate chronic inflammatory diseases [10]. Therefore, distinguishing between different types of adipose tissue is crucial for understanding and stratifying IBD [9]. Some studies have proposed a visceral adipose tissue (VAT) to subcutaneous adipose tissue (SAT) ratio as a means to better stratify CD severity in individuals [11, 12].

Recent studies have demonstrated the accurate measurement and quantification of adipose tissue distribution, particularly the “visceral fat area” (VFA), and muscle mass to assess the grade of myopenia using computed tomography (CT) or magnetic resonance imaging (MRI), and the strong correlation with the visceral fat mass [13–15].

However, there is currently a limited number of studies investigating the impact of VFA in CD patients. While immunosuppressive medication is becoming increasingly effective in treating IBD, a significant proportion of CD patients still undergo abdominal surgery at least once during their lifetime [16]. Ileocecal resection (ICR) remains the most common surgical procedure and has been associated with longer relapse-free survival in ileocecal CD compared to initial immunosuppressant medication [3, 17]. Although some data suggest that microscopic inflammation in ileocecal resections does not significantly increase the risk of anastomotic leakage [18], the impact of preoperatively measured visceral fat on postoperative complications and CD relapse has not been definitely established.

Given the scarcity of data in this area, the objective of the present study is to assess the influence of MRI-measured VFA on the postoperative course following ICR for CD. Due to a lack of data, the present study was designed to evaluate the influence of MRI-measured VFA on the postoperative course following ileocecal resection for CD. Our hypothesis was that the amount of visceral fat could serve as an indicator for development of anastomotic leakage in these patients.

## Material and methods

### Study design and participants

We conducted a retrospective review of 347 consecutive patients aged 18 years or older who underwent ICR for CD at our tertiary referral center for IBD between 2010 and 2020.

A total of 223 patients with available and high-quality preoperative MRI scans, conducted within 12 months prior to ICR, were included in the study. All included patients received preoperative up-to-date radiological cross-sectional imaging. Only patients were included who had also undergone high quality MRI imaging within the last 12 months before surgery, and whose findings from this examination closely matched those of the current preoperative imaging. Patients who had additional synchronous bowel resection related to CD or underwent ICR for indications other than CD were excluded. Indications for ileocecal resection included post-inflammatory fibrotic strictures and a fistulizing disease with or without abscess. Patients younger than 18 years of age were also excluded. We recorded patient demographics, postoperative outcomes, and complications, and recurrence rates. Using an established image-analysis method, we measured total fat area (TFA), visceral fat area (VFA), subcutaneous fat area (SFA), and abdominal circumference (AC) on MRI cross-sectional images at the lumbar 3 (L3) level. Additionally, we calculated ratios based on these values.

All ICRs were performed according to internal clinic standards. Based on the surgical indication, the operation was either performed laparoscopically, using three laparoscopic access ports and either a minilaparotomy in the right lower quadrant, or a small midline incision, or conventionally with a midline laparotomy. The extent of the resection involved the terminal ileum and the cecum. The resection margins were determined by the surgeon to be in macroscopically unaffected tissue. Until January 2020, the anastomoses were created as stapled or hand-sewn antimesenteric side-to-side anastomoses. Since January 2020, the Kono-S-anastomosis [19] has been introduced as the standard procedure. Each patient received intravenous antibiotic prophylaxis before surgery. Postoperative care and management followed established protocols for ICR. All patients received a postoperative IBD-specific consultation by gastroenterology specialists, in which, in accordance with German S3 IBD guidelines, the use of anti-TNF-alpha antibodies was recommended for maintaining remission. Medication therapy was tailored based on the previously administered medication, taking into account any interim occurrences of intolerance or loss of efficacy.

Demographic information, intraoperative findings, postoperative complications, and recurrence rates were collected from the hospital´s electronic health records system. The analysis included variables such as gender, age, length of surgery, ASA (American Society of Anesthesiologists) score, body mass index (BMI), other systemic pre-existing diseases, immunosuppressive medication, preoperative hemoglobin value, type of anastomosis, surgical technique, intraoperative bowel diversion, degree of inflammation at the resection margins, use of opiates, peridural anesthesia, postoperative use of neostigmine, metoclopramide, bisacodyl, enemas, parecoxib, anastomotic leak, complication rate (according to the Clavien-Dindo classification [20]), length of hospital stay and postoperative endoscopic or clinical recurrence rates of CD at the anastomosis.

The primary endpoint of the study was the incidence of postoperative anastomotic leakage at the ileocolonic anastomosis within 30 days postoperatively, which was diagnosed using radiological imaging, laboratory tests and clinical evaluation. Secondary endpoints included postoperative complications assessed according to the Clavien-Dindo classification within 30 days postoperatively and clinical CD recurrence at the anastomosis site. CD recurrence was defined as the reappearance of the clinical manifestations of CD that required medical or surgical treatment, as confirmed by endoscopy according to the Rutgeerts score [21] (i2 or higher), radiological evidence of disease, or histological evidence after surgery. In order to assess postoperative CD recurrence rates, patients were followed up with endoscopies on a regular basis. The endoscopies were carried out regularly and as needed in the first year after the operation followed by annual endoscopies at the Gastroenterology Outpatient Clinic.

The study protocol was approved by the Medical Ethical Committee of Charité – Universitätsmedizin Berlin (EA4/148/20).

### MR image analysis

Images were retrieved from digital storage in the local Picture Archiving and Communication System (PACS). If patients had undergone multiple MRIs within the 12 months preceding surgery, only the most recent one was included for analysis. A board-certified radiologist screened all MRI scans before analysis. Data sets with poor image quality, significant artifacts such as motion or implant-related artifacts, or incomplete axial depiction of the abdominal wall at level L3were excluded from the analysis, resulting in the final cohort of 223 subjects, after excluding 30 cases.

MR image analyses were conducted using the Visage^®^7 software (Visage Imaging GmbH, Berlin, Germany). Based on axial T1 sequences without fat suppression or DIXON technique fat only sequences, several parameters were measured semi-automatically at the level of the third lumbar vertebra (L3):abdominal circumference (AC, in cm), total fat area (TFA, in cm^2^),visceral fat area (VFA, in cm^2^), and subcutaneous fat area (SFA, in cm^2^). When multiple slices at L3 were available, the slice offering the best visibility of both transverse processes was selected. According to Shen et al. [22, 23], the single slice VFA measurement at level L3 showed the strongest correlation with the overall volume of visceral adipose tissue.

**Fig. 1:**
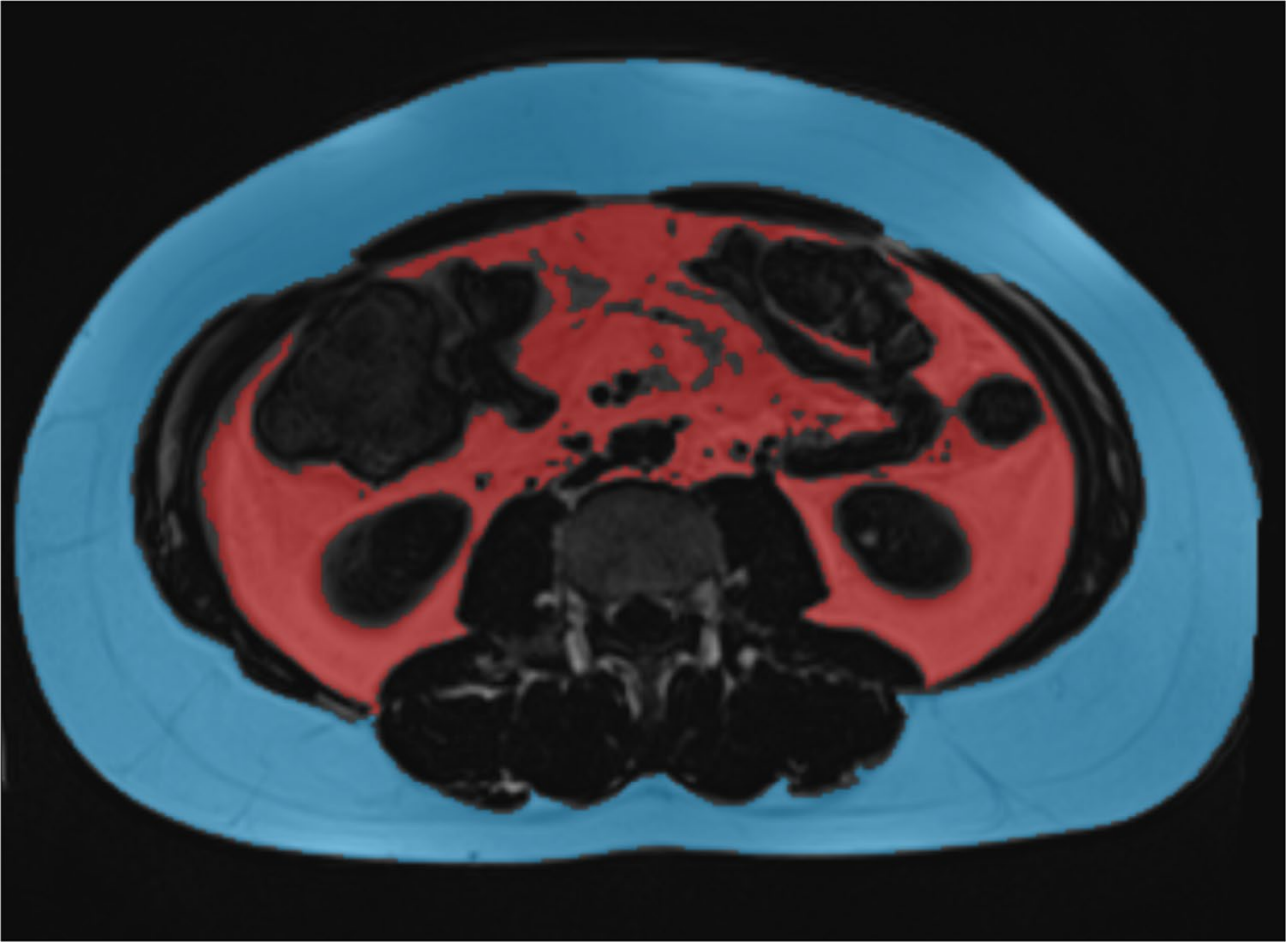
Display of Visceral Fat Area (VFA, red) and Subcutaneous Fat Area (SFA, blue)

To measure TFA, a mask encompassing all pixels with a signal intensity above a manually set threshold, indicative of adipose tissue was created. In a subsequent step, intra- and intermuscular fat tissue, as well as fat signal equivalents (e.g. within the colon), were manually deselected. VFA was calculated by limiting the region of interest to the intraabdominal area. Finally, SFA was derived by subtracting TFA from VFA. Figure 1 illustrates an example of VFA and SFA. Additionally, to account for variations in patients´ body height, a VFA/SFA ratio (V/S ratio), total fat index (TFI = TFA/m^2^), subcutaneous fat index (SFI = SFA/m^2^), and visceral fat index (VFI = VFA/m^2^) were.

All image analyses were conducted by a single researcher who remained blind to the patients´ information.

### Statistical analysis

Categorical variables were presented as percentages (%). Quantitative variables were presented as median ± standard deviation (SD). To compare continuous variables the Mann-Whitney U test was used. Categorical variables were compared using the chi-square test. Fisher’s exact test was utilized for analyzing categorical data when the sample size was small. Univariable analysis was used for our primary endpoint anastomotic leakage and following secondary endpoints: rates of overall postoperative complications, reoperation, length of hospital stay, and CD recurrence. Only significant variables from the univariable analysis were entered into the multivariable regression model. A p-value of ≤ 0.05 was considered statistically significant. For secondary endpoints, p-values were considered exploratory and presented without Bonferroni correction. Statistical analysis was performed using SPSS Statistics Software 25.0 (IBM, Armonk, NY, USA).

## Results

### Patient characteristics

The study included 223 patients who underwent ICR for CD. A total of 223 patients were included in the study with 116 (52%) being female and 107 (48%) being male. The majority of patients (n = 202, 90.6%) had an ASA classification of I and II and 21 of III and IV. 63.2% (n = 141) of the patients underwent surgery while on immunosuppressive medication. Specifically, 8.5% (n = 19) were taking 5-aminosalicylic acid, 28.3% (n = 63) were on steroids, 21.5% (n = 48) were taking Azathioprine, and 22.9% (n = 51) were receiving monoclonal antibodies. Among the patients, 16.2% (n = 28) were active smokers, 78% (n = 135) were non-smokers and 5.8% (n = 10) were formally smoking. Laparoscopic surgery was performed in 68.5% (n = 152) of cases, of which 12.6% (n = 28) of these cases required conversion from laparoscopic to an open approach and 18.9% (n = 42) were operated on openly. A protective ileostomy was created in 24.7% (n = 55) of cases.

VFA was significantly higher in patients aged 35 years or older (p < 0.001) and in patients with preexisting vascular disease (p < 0.001). Male patients also exhibited a significantly higher VFA (p < 0.007). No differences in VFA and anemia, immunosuppressive medication or current smoking status were found in between both age groups. Visceral fat index (VFI) was significantly higher in patients aged 35 years or older (p < 0.001), those with ASA III-IV (p = 0.015) and patients with preexisting vascular disease (p < 0.001). Patients who underwent open surgery as the primary approach demonstrated a significantly higher VFA (p = 0.030) and VFI (p = 0.008) (Table 1).

**Table 1:** Patient characteristics subject to ileocecal resection for Crohn´s disease in correlation with visceral obesity

**Variable**		**Visceral obesity**			
		**Visceral fat area**		**Visceral fat index**	
		**(VFA) [cm**^**2**^**]**		**(VFI) [cm**^**2**^**/m**^**2**^**]**	
	*n* = 223	median	*P* value	median	*P* value
**Preoperative**					
Age, years	35 (26 – 48)				
≤ 35	114 (51.1)	39.6	** < 0.001**	13.7	** < 0.001**
		(25.6–58.7)		(88.5–19.2)	
> 35	109 (48.9)	89.8		29.6	
		(45.6–155.9)		(15.9–51.4)	
Sex					
Female	116 (52)	44.2	**0.007**	17.1	0.078
		(33.8–81.9)		(12.2–28.0)	
Male	107 (48)	69.6		22.5	
		(32.7–136.8)		(10.3–45.9)	
ASA score					
I–II	202 (90.6)	48.6	0.186	17.7	**0.015**
		(33.6–97.3)		(11.6–34.2)	
III–IV	21 (9.4)	82		29.7	
		(30.8–150.0)		(20.2–51.5)	
Vascular disease					
Yes	30 (13.5)	119.3	** < 0.001**	40.6	** < 0.001**
		(87.7–179.2)		(27.8–62.9)	
No	192 (86.1)	46.5		16.1	
		(29.7–85.9)		(10.8–29.1)	
Kidney failure					
Yes	23 (10.3)	71.6	0.683	21.3	0.963
		(27.3–135.0)		(9.7–42.9)	
No	191 (85.7)	49.1		17.7	
		(33.2–98.5)		(11.8–35.1)	
Anemia (Hb, g/dl)					
≤ 12	91 (40.8)	56	0.496	18.5	0.9
		(33.6–113.1)		(11.4–36.9)	
> 12	126 (56.5)	49.6		17.7	
		(31.5–90.5)		(11.8–30.8)	
Immunosuppression	141 (63.2)	51.1		17.9	
		(34.7–97.3)		(12.8–34.3)	
5-ASA	19 (8.5)	68.4	0.178	24.9	0.132
		(41.8–125.2)		(14.6–48.7)	
Steroids	63 (28.3)	64.3	0.188	22.6	0.226
		(37.8–99.0)		(13.8–35.1)	
Azathioprine	48 (21.5)	43.4	0.154	14.7	0.112
		(30.5–79.8)		(12.0–25.1)	
Monocl. antibody	51 (22.9)	51.1	0.854	17.9	0.71
		(36.6–98.3)		(11.6–34.4)	
Nicotine					
Yes	28 (16.2)	63.2	0.185	24.6	0.138
		(32.7–98.9)		(14.3–35.7)	
No	135 (78.0)	54.4		18.7	
		(33.2–99.8)		(11.7–36.0)	
Ex-smoker	10 (5.8)	99.4		34.2	
		(52.9–147.3)		(21.6–46.8)	
**Intraoperative**					
Surgical approach					
Laparoscopic	152 (68.5)	48.1	**0.03**	17.8	**0.008**
		(29.0–95.3)		(10.3–33.8)	
Conversion	28 (12.6)	41.8		13.8	
		(34.4–89.6)		(12.0–32.0)	
Open	42 (18.9)	69.6		25.2	
		(41.7–137.8)		(15.9–48.2)	
Operation time, min	150 (129–193)				
≤ 150	111 (49.8)	47	0.053	16.4	0.118
		(28.0–90.5)		(10.8–30.8)	
> 150	110 (49.3)	61.4		22.1	
		(36.2–108.9)		(12.6–37.0)	
Ileostomy					
Yes	55 (24.7)	58.5	0.277	20.3	0.131
		(36.9–102.5)		(13.7–40.4)	
No	168 (75.3)	48.6		17.8	
		(31.5–98.3)		(11.4–33.8)	

Bolded numbers indicate significant results, respectively results with a clear trend towards significance

Data are described as n (%) or median (IQR)

*ASA* American Society of Anesthesiology, *Hb* hemoglobin, *5-ASA* 5-aminosalicylic acid

### Surgical outcome and recurrence at the anastomosis

In our cohort, a total of 26 patients (11.7%) experienced anastomotic leakage with 15 (57.7%) being female and 11 (42.3%) being male. No significant difference could be shown between both sexes (p = 0.677). Postoperative complications, as indicated by Clavien-Dindo-Score I-V, were observed in 28.7% of the patients. Among them, 19.8% required reoperation due to postoperative complications. The median follow-up time for all patients was 48.8 months (IQR: 20–82.9 months) and 8.1% of them developed clinical recurrence after a median time of 10.2 months (IQR: 5.7–15.4) with 13 (76.5%) being female and 4 (23.5%) being male. There was no difference in length of hospital stay (LOS) based on VFA or VFI (Table 2).

**Table 2:** Surgical outcomes and recurrence of Crohn’s disease in correlation with visceral obesity

**Variable**		**Visceral obesity**			
		**Visceral fat area**		**Visceral fat index**	
		**(VFA) [cm**^**2**^**]**		**(VFI) [cm**^**2**^**/m**^**2**^**]**	
	*n* = 223	median	*P* value	median	*P* value
Anastomotic leak					
Yes	26 (11.7)	60.8 (29.4–100.7)	0.982	18.5 (11.5–32.0)	0.684
No	196 (88.3)	50.1 (33.6–99.9)		18.0 (11.9–35.3)	
Any Complication Clavien Dindo grade I-V					
Yes	64 (28.7)	58.1	0.885	18.5	0.834
		(30.1–105.9)		(11.5–36.1)	
No	159 (71.3)	49.4		17.9	
		(33.8–98.6)		(11.9–34.8)	
Reoperation					
Yes	44 (19.8)	58.1 (28.7–97.5)	0.839	18.5 (10.9–29.7)	0.461
No	178 (80.2)	50.1 (33.9–101.1)		18.3 (12.1–35.5)	
LOS, days	8 (6 – 11)				
LOS ≤ 8	126 (56.5)	47.4	0.147	16.4	0.224
		(32.3–95.2)		(11.7–33.7)	
LOS > 8	96 (43.0)	58.1		20.7	
		(34.6–123.6)		(11.4–40.6)	
CD recurrence					
Yes	18 (8.1)	47.7	0.916	18.8	0.912
		(38.1–90.9)		(12.2–33.2)	
No	204 (91.9)	51.1		18.3	
		(32.6–102.2)		(11.6–35.3)	

Data are described as n (%) or median (IQR)

*LOS* length of stay

The median VFA did not differ significantly in the group of anastomotic leakages (p = 0.470) nor in the group of CD recurrences (p = 0.906). Likewise, there was no significant association between abdominal circumference (AC) (p = 0.907), total area (TA) (p = 0.767), TFA (p = 0.932), total fat index (TFI) (p = 0.783), SFA (p = 0.951), SFA/TFA ratio (p = 0.422), subcutaneous fat index (SFI) (p = 0.986), VFA (p = 0.982), VFA/SFA ratio (p = 0.422), VFA/TFA ratio (p = 0.422), visceral fat index (VFI) (p = 0.684), and the occurrence of anastomotic leakage (Table 3). Furthermore, no significant association was found between AC (p = 0.754), TA (p = 0.693), TFA (p = 0.264), TFI (p = 0.282), SFA (p = 0.103), SFI (p = 0.141), VFA (p = 0.916), VFI (p = 0.912) and clinical CD recurrence at the anastomosis (Table 3). A subgroup analysis for female and male patients separately showed no association between visceral obesity parameters (VFA or VFI) and anastomotic leak or CD recurrence (Table 4). A slight trend was observed between SFA/TFA ratio (p = 0.059), VFA/TFA ratio (p = 0.059), VFA/SFA ratio (p = 0.059), and the occurrence of a CD relapse (Table 3).

**Table 3:** Overview of radiologic parameters for body fat distribution in correlation with anastomotic leakage and recurrence of Crohn’s disease

	**Anastomotic leakage**			**Recurrence**		
	Yes (n = 26)	No (n = 196)	*P* value	Yes (n = 18)	No (n = 204)	*P* value
BMI [kg/m^2^]	21.3 (19.7–23.5)	21.8 (19.1–24.7)	0.470	22.1 (20.8–22.8)	21.5 (19.1–24.7)	0.906
AC [cm]	83.8 (76.9–100.1)	84.7 (77.2–94.5)	0.907	84.6 (78.8–95.7)	84.8 (77.0–94.7)	0.754
TA [cm^2^]	502.2 (438.3–677.9)	509.4 (434.9–635.8)	0.767	504.6 (445.4–660.8)	509.4 (426.3–643.5)	0.693
Total fat area (TFA) [cm^2^]	170.5 (114.2–316.3)	178.2 (93.8–277.6)	0.932	215.1 (140.4–344.7)	175.4 (93.4–272.6)	0.264
TFI [cm^2^/m^2^]	57.9 (34.3–95.0)	60.8 (31.4–99.1)	0.738	71.4 (44.2–119.1)	59.6 (31.0–95.9)	0.282
**Subcutaneous obesity**						
Subcutaneous fat area (SFA) [cm^2^]	102.2 (69.2–184.3)	104.2 (55.8–165.5)	0.951	149.7 (91.5–233.6)	103.7 (55.8–163.6)	0.103
SFA/TFA ratio	0.684 (0.598–0.748)	0.643 (0.523–0.741)	0.422	0.729 (0.650–0.782)	0.643 (0.523–0.741)	0.059
SFI [cm^2^/m^2^]	38.4 (20.3–63.3)	36.4 (18.9–58.0)	0.986	49.4 (26.6–84.3)	36.1 (18.9–57.5)	0.141
**Visceral obesity**						
Visceral fat area (VFA) [cm^2^]	60.8 (29.4–100.7)	50.1 (33.6–99.9)	0.982	47.7 (38.1–90.9)	51.1 (32.6–102.2)	0.916
VFA/SFA ratio	0.462 (0.337–0.672)	0.555 (0.350–0.912)	0.422	0.372 (0.278–0.539)	0.555 (0.350–0.912)	0.059
VFA/TFA ratio	0.316 (0.252–0.402)	0.357 (0.259–0.477)	0.422	0.271 (0.218–0.350)	0.357 (0.259–0.477)	0.059
VFI [cm^2^/m^2^]	18.5 (11.5–32.0)	18.0 (11.9–35.3)	0.684	18.8 (12.2–33.2)	18.3 (11.6–35.3)	0.912

Data are described as n (%) or median (IQR)

*BMI* body mass index, *AC* abdominal circumference, *TA* total area in axial scanning view; total fat area (TFA = SFA + VFA), *TFI* total fat index (TFA divided by the square of height), *SFI* subcutaneous fat index (SFA divided by the square of height), *VFI* visceral fat index (VFA divided by the square of height)

**Table 4:** Anastomotic leak and recurrence of Crohn’s disease in correlation with visceral obesity for female and male patients

Variable		**Visceral obesity**			
		**Visceral fat area**		**Visceral fat index**	
		**(VFA) [cm**^**2**^**]**		**(VFI) [cm**^**2**^**/m**^**2**^**]**	
		median	*P* value	median	*P* value
**Female**	*n* = 116	44.2 (33.8–81.9)		17.1 (12.2–28.0)	
Anastomotic leak					
Yes	15 (12.9)	41.4 (28.4–90.9)	0.59	16.1 (11.4–29.7)	0.609
No	101 (87.1)	44.2 (34.3–81.9)		17.2 (12.6–27.9)	
CD recurrence					
Yes	13 (11.2)	47.7 (38.1–77.6)	0.555	18.8 (14.5–27.0)	0.601
No	103 (88.8)	43.2 (32.6–83.7)		16.4 (12.0–29.3)	
**Male**	*n* = 106	69.6 (32.7–136.8)		22.5 (10.3–45.9)	
Anastomotic leak					
Yes	11 (10.4)	86.6 (41.7–144.5)	0.586	23.5 (10.8–45.4)	0.981
No	95 (89.6)	63.2 (30.9–136.8)		22.3 (10.3–46.3)	
CD recurrence					
Yes	4 (3.8)	128.6 (18.4–222.1)	0.714	38.2 (5.5–74.8)	0.786
No	101 (96.2)	66.7 (31.7–136.1)		22.1 (10.3–45.7)	

Data are described as n (%) or median (IQR)

A subgroup analysis examining differences in VFA and VFI based on the severity of postoperative complications, according to the Clavien Dindo classification, revealed no significant differences (Kruskal–Wallis Test for VFA and VFI: p = 0.559 and p = 0.213, respectively).

## Discussion

Given the complex nature of CD and its various manifestations, this study aims to investigate whether preoperative radiologically measured mesenteric fat at level L3 can reliably predict anastomotic leakage, postoperative complications, or CD recurrence in patients with defined CD, specifically limited to ileitis terminalis.

Anastomotic leakage rates in CD are generally higher than in other bowel resections, with reported rates ranging from 6.4–14% [24]. In our group, we observed an anastomotic leakage rate of 11.7%, consistent with current literature in Crohn's surgery. Anastomotic leakage can significantly increase the likelihood of CD recurrence at the anastomosis [25]. However, in our patient cohort, with a leakage rate of 11.7% and a recurrence rate at the anastomosis of 8.1% within a median of 10.2 months (IQR: 5.7–15.4) after surgery, this does not appear to have an impact. Furthermore, in our patient cohort, we could not demonstrate a significant association between any of the radiologically measured parameters and the occurrence of anastomotic leakage. There is currently no evidence in the literature on this question, which is why prospective studies should be conducted in the future to verify these findings.

The influence of cachexia or malnutrition on postoperative complications plays a crucial role, especially in IBD. Many patients are in a poor nutritional state at the time of surgery due to constant diarrhea, bowel stenosis, abdominal pain, and the disease activity itself. A deficiency of proteins can hinder postoperative healing and weaken the immune system [26]. Tjeertens et al. demonstrated in a general surgery patient population that while obese patients have a higher immediate risk of complications and longer surgery duration, overall survival is worse in cachectic patients [6]. Obesity plays a significant role in the development of postoperative surgical site infections and other minor complications [27], but it does not significantly impact the development of major postoperative complications [28]. Notably, there were no morbidly obese patients in our patient population. However, a significant difference in VFA and VFI was observed between the group “ < 35 years” and the group “ ≥ 35 years”. Watanabe et al. showed in colon cancer patients that visceral adiposity, expressed by VFA, is a better predictor of postoperative complications than BMI [29]. Still, the findings of that study cannot be applied to our patient population as the research group categorized patients into “non-obese” with a VFA of < 100 cm^2^ and „obese “ with a VFA of > 100 cm^2^. The median VFA in both age groups in our study was well below the cut-off value for “obese/non-obese”, with 39.6 cm^2^ (< 35 years) and 89.8 cm^2^ (≥ 35 years). While we observed a significant difference in the volume of fat distribution between the two groups, it did not have an impact on the likelihood of postoperative complications. It is possible that a volume of < 100 cm^2^ is too small to have an influence on potential complications. Furthermore, IBD has a different pathomechanism than colon carcinoma. In patients with colorectal cancer and obesity, there is a general visceral adiposity that that can lead to postoperative complications due to poorer microvascularization. In contrast, IBD patients typically experience localized increase in visceral fat due to locally confined inflammatory reactions.

A research group led by Catherine Rowan showed in a large meta-analysis from 2021 that increased visceral fat in CD patients may be associated with a more severe course of disease and a more complex CD phenotype. This study also suggested an increased recurrence rate after surgery in patients with increased visceral fat. However, the question of postoperative complications and their association with visceral fat could not be conclusively answered due to poor data quality and low case numbers in the studies used for this meta-analysis [9]. Our data do not provide evidence of a significant clustering of complications in relation to increased visceral fat. In contrast to the studies included in the meta-analysis by Rowan et al., our cohort includes a larger number of patients, likely providing more reliable data. In our patient population, no increased recurrence rate based on the volume of visceral fat was observed. All patients included in our study received a side-to-side anastomosis (conventional stapled or hand-sewn) or Kono-S-anastomosis, which are always wider than end-to-end anastomoses. Generally, for CD patients, the creation of side-to-side anastomoses is recommended, as they have a lower restenosis rate over time [25]. However, in the studies included in the meta-analysis, no conclusion can be drawn regarding the type of anastomoses used. Therefore, the studies may have also included larger numbers of end-to-end anastomoses in the data collection. Furthermore, in most studies included in this meta-analysis, patients were only followed up for possible anastomotic restenosis for 6 months postoperatively. In our study, the median follow-up period was 48.8 months (IQR: 20–82.9 months). Therefore, based on our data, the statement, that visceral fat in CD has no influence on postoperative complications and CD recurrence at the anastomosis is reliable due to a larger number of patients, standardized anastomosis techniques, and a longer follow-up period.

Li et al. also investigated the influence of visceral fat on complications and the recurrence rate at the anastomosis in CD patients. This research group found a recurrence rate of 66.6% at the anastomosis 6 months after surgery in patients with increased VFA [30]. In our patient population, we observed a recurrence rate of only 8.1%, during a median follow-up time of 48.8 months (IQR: 20–82.9 months). after surgery. The difference in case numbers may play a role (Li et al. n = 72 vs. n = 223 in the present study). Additionally, the study does not provide any conclusions about the type of anastomosis, raising the possibility that different anastomotic techniques, possibly not appropriate for the underlying disease, were performed, which naturally entail a higher recurrence rate.

While we could not establish a significant association between all radiologically measured parameters and CD recurrence at the anastomosis, a statistical trend is evident between SFA/TFA ratio, VFA/SFA ratio and VFA/TFA ratio, and the occurrence of a CD relapse. As increased visceral fat, as demonstrated by Rowan et al., reflects a more severe course of disease [9], it is possible that these patients may develop a recurrence over time more often than other patients with lower fat ratios. To conclusively address this point, future studies should extend the follow-up period accordingly.

## Limitations

This study has several limitations, as is common in retrospective studies. Despite its retrospective design, this study benefits from analyzing a homogeneous group with respect to underlying disease, surgical and medical therapy, and gender distribution. However, only patients who underwent ICR for CD and had high-quality preoperative MRI scans available were included in this study. Nevertheless, given the large number of patients enrolled during the study period, when compared to the disease´s incidence and prevalence, as well as similar studies, it is unlikely that selection bias exists with 223 included patients. Potential biases could result from emergency surgeries where patients may not have received appropriate preoperative imaging and fall outside the scope of our analysis concerning VFA, VFI, and postoperative complications. However, since very few CD patients undergo absolute emergency procedures without appropriate imaging, this bias is unlikely.

Certainly, the timeframe of preoperative MRI imaging poses a certain limitation. However, the choice of the inclusion period, especially in retrospective analyses, always represents a balance between practicability, standardization, data availability, clinical relevance, and the need for the most current data. While the choice of a 12-month window for MRI imaging may be debatable, the study´s findings stand independently based on the reported results and statistical analysis.

Another limitation may pertain to the assessment of CD relapse at the anastomosis. The development of a CD relapse is a multifactorial process and depends, among other factors, not only on the pharmacological relapse prophylaxis but also on the width of the anastomosis, postoperative complications at the anastomosis, and the time elapsed since the operation. Since we routinely perform antimesenteric side-to-side anastomoses or Kono-S-Anastomoses, the likelihood of early stenosis due to overly tight anastomoses is minimal. As for the follow-up period, it would certainly be useful to follow up these patients for a longer period in future studies. While a longer follow-up period would be valuable in future studies, it is worth noting, that a 6-months follow-up period is commonly employed in the literature to evaluate anastomotic recurrence following surgery. Therefore, our data provides a valid comparison to other studies.

Another limitation of this study lies in the nature of a retrospective analysis and the fact that both medical and surgical therapies and techniques have evolved over the course of 10 years. Patients operated on since 2020 underwent reconstruction using a Kono-S-anastomosis a technique developed in recent years for ICR in CD, which reduces the likelihood of early restenosis at the anastomosis. However, the positive influence of this reconstruction on Crohn´s relapse at the anastomosis has not been definitely established and is subject to current investigations [31]. Therefore, our data, despite the variation in anastomotic reconstructions within the cohort, yields consistent results concerning recurrence.

## Conclusion

In the largest and most homogeneous cohort of CD patients who underwent ICR and had high-quality pre-surgery MRIs, we have found no evidence to support an association between either visceral or subcutaneous fat and anastomotic leakage, postoperative complications, or clinical recurrence at the anastomosis. While measuring visceral fat may serve as a useful parameter for assessing the severity of CD activity, it appears to lack specificity in predicting potential postoperative complications and CD relapse.

## Data Availability

The data supporting the findings of this study are available from the corresponding author upon reasonable request.
